# Social support and subsequent cognitive frailty during a 1-year follow-up of older people: the mediating role of psychological distress

**DOI:** 10.1186/s12877-022-02839-5

**Published:** 2022-02-28

**Authors:** Yi Wang, Jie Li, Peipei Fu, Zhengyue Jing, Dan Zhao, Chengchao Zhou

**Affiliations:** Centre for Health Management and Policy, School of Public Health, Cheeloo College of Medicine, Shandong University; NHC Key Laboratory of Health Economics and Policy Research, Shandong University, 44 Wen-hua-xi Road, Jinan, 250012 Shandong China

**Keywords:** Social support, Cognitive frailty, Psychological distress, Longitudinal study, Older people

## Abstract

**Background:**

Frailty and cognitive impairment are two common geriatric symptoms linking adverse health-related outcomes. However, cognitive frailty, a new definition defined by an international consensus group, has been shown to be a better predictor of increased disability, mortality, and other adverse health outcomes among older people than just frailty or cognitive impairment. This study estimated the prospective association between social support and subsequent cognitive frailty over 1 year follow-up, and whether psychological distress mediated the association.

**Methods:**

The data was drawn from a prospective repeated-measures cohort study on a sample of participants aged 60 and over. A total of 2785 older people who participated in both of the baseline and 1-year follow-up survey were included for the analysis. Cognitive frailty was measured by the coexistence of physical frailty and cognitive impairment without dementia. Control variables included sex, age, education, marital status, economic status, smoking status, alcohol drinking status, chronic conditions, and functional disability. Path analyses with logistic function were performed to examine the direct effects of social support (predictors) on subsequent cognitive frailty (outcome) at 1-year follow-up and the mediating role of psychological distress (mediator) in this link.

**Results:**

After adjusting for covariates and prior cognitive frailty status, social support was negatively associated with psychological distress (β = − 0.098, 95% CI = − 0.137 to − 0.066, *P* < 0.001) and was negatively associated with the log-odds of cognitive frailty (β = − 0.040, 95% CI = − 0.064 to − 0.016, *P* < 0.001). The magnitude of mediation effects from social support to cognitive frailty via psychological distress was a*b = − 0.009, and the ratio of a*b/(a*b + c’) was 24.32%.

**Conclusions:**

Lower social support is associated with increased rates of subsequent cognitive frailty over 1-year follow-up, and this link is partially mediated through psychological distress, suggesting that assessing and intervening psychological distress and social support may have important implications for preventing cognitive frailty among older people.

## Background

Frailty and cognitive impairment are two common geriatric symptoms linking adverse health-related outcomes [1]. However, cognitive frailty, a new definition proposed by the International Academy on Nutrition and Aging (I.A.N.A) and the International Association of Gerontology and Geriatrics (I.A.G.G) in 2013, was defined as “a heterogeneous clinical condition characterized by the simultaneous presence of both physical frailty and cognitive impairment and the exclusion of concurrent Alzheimer disease or other dementias” [2]. Increasing studies have shown that cognitive frailty plays a better role in predicting the short-term and the long-term all-cause mortality, disability, dementia, and other adverse health outcomes among older people than just frailty or cognitive impairment [3–6]. Importantly, the condition of cognitive frailty could recovery physically robust and/or cognitively normal due to its reversible process if effective interventions were employed [7]. Therefore, it is imperative to identify predictive and modifiable risk factors and underlying mechanisms concerning cognitive frailty in order to inform intervention strategies among older people. However, to date, the possible determinants as well as the underlying mechanisms of cognitive frailty among older people are still poorly understood.

One potential modifiable protective factor for cognitive frailty among older people is social support [8], which is defined as “the perceived and actual assistance that individuals can receive from family, friends, and other connections in the social environment” [9]. Many studies have shown that social support is associated with increased risk of both physical frailty and cognitive impairment among older people [10, 11]. However, previous studies on the association between social support and cognitive frailty are predominantly cross-sectional [12, 13], we found no prospective study specifically examined the longitudinal association between social support and subsequent cognitive frailty among older people in rural China. Identifying mediating factors could be important to further understand the relationship between social support and cognitive frailty. The psychological process driving the link between social support and cognitive frailty is not clear. Previous studies in other countries have shown that psychological health is associated with both of cognitive frailty [6] and social support [14], suggesting that the association between social support and cognitive frailty might be through psychological mechanism. While prior studies have investigated the direct effects of both social support and psychological health on cognitive frailty separately, no study has investigated potential mediating effects of psychological health on the link between social support and cognitive frailty among older people. In addition, previous studies have shown that psychological health plays an important mediating role in relationships between some factors and health [15, 16]. A recent study has shown that the relationship between cognitive function and physical frailty is partially mediated by psychological distress [17]. Consequently, one of the underlying mechanisms between social support and cognitive function might be through psychological distress.

Using the longitudinal data from the Shandong Rural Elderly Health Cohort (SREHC), the current analysis was conducted to understand the link between social support and subsequent cognitive frailty over 1-year follow-up. Specifically, our first aim, as part of the search to prove the main hypothesis (the second aim), was to examine whether lower levels of social support increased the risk of cognitive frailty among older people during the subsequent year. As our main objective, the second aim was to examine the main hypothesis that psychological distress meditated associations of social support with cognitive frailty.

## Methods

### Study design and participants

SREHC is a longitudinal study of older people behavior and health in rural Shandong, China. We used stratified multistage sampling to select our participants [18]. To begin this process, all counties of Shandong province were categorized into three levels (low, medium, and high) based on the GDP per capita in 2018. The second step was to select one rural county randomly in each level, and Rushan, Qufu, and Laoling were selected as our study sites. Once the county was chosen, five townships were chosen from each aforesaid county randomly. Third, four communities/villages were chosen from each township randomly. In each community/village, we chose individuals aged 60 years or over using village resident registry randomly. A total of 3243 respondents aged 60+ without a clinical diagnosis of dementia and psychiatric diseases participated the baseline survey from May 2019 to June 2019. Of the 3243 respondents at baseline, 2785 participated in the follow-up survey from August 2020 to September 2020, with a follow-up rate of 85.88%. To ensure quality, both the two surveys were conducted by the same group of trained master public health students using the same questionnaires face-to-face. Training was supervised by our principal investigator, and all questionnaires were double-checked by our quality group. Our trained students read the questionnaires to the poor vision of older individuals. Before each survey, we obtained the written informed consents from each respondents stating the study purposes, value, methods, and potential risks. For illiterate older people, in addition to obtaining their verbal consent, we also require their legally/kin authorized representative to provide a proxy written informed consent. This study was reviewed and approved by the Ethics Committee of Shandong University (approval No. 20181228) in accordance with the Declaration of Helsinki.

### Cognitive frailty

According to the definition from I.A.N.A and I.A.G.G, respondents who had the co-existence of physical frailty and cognitive impairment were classified as having cognitive frailty in this study. Specifically, cognitive impairment was assessed by the Chinese version of the Mini Mental Status Examination (MMSE) [19]. MMSE has been widely used in the assessment of cognitive function level in older adults and has excellent reliability and validity [20]. The cut-off values of the MMSE for cognitive impairment according to educational level were ≤ 17 for the uneducated, ≤ 20 for the primary school educated, ≤ 22 for those accepted the junior high school, and ≤ 24 for those university or above, respectively [13]. Most of the measures of cognitive frailty use Fried frailty phenotype [21, 22] because it is more suitable for an immediate identification of non-disabled elders at risk of negative events. Thus, in our study, frailty was measured by frailty phenotype criteria, which was proposed and validated by Fried et al. [23]. Frailty phenotype consists of 5 items: shrinking (unintentional weight loss), weakness (grip strength), self-reported exhaustion, slowness (a walking time of 15 ft adjusted by gender and height), and low activity. Older people with 3-5 criteria were considered frail.

### Social support

We adopted the Social Support Rating Scale (SSRS) [24] to measure social support. The SSRS is a 10-item self-reported scale composed of three parts: subjective support, objective support, and support utilization. Subjective support reflects perceived social support that individuals feel understood, supported, or helped by others. Objective support represents the actual support that individuals received, such as the financial support and the practical assistance. The utilization of support reflects the degree of social support used, such as individuals how to seek and get actual help when in need. The SSRS has been shown a reliability and validity measure in China [25, 26], The total score of SSRS ranges from 12 to 66, with higher scores indicate higher levels of social support.

### Psychological distress

The Kessler Psychological Distress Scale (K10) was adopted to identify the non-specific psychological distress in this study, including depression and anxiety disorders [27, 28]. The K10 has been confirmed to have high reliability and validity in China [17, 29]. It contains 10-item and each item is assessed by using a 5-point Likert-type from 1 (none of the time) to 5 (all the time). The possible score ranges from 10 to 50, with a higher score indicating higher levels of psychological distress.

### Potential confounders

We identified potential confounders for social support and cognitive frailty based on the previous studies [12, 30, 31]. Potential confounders included socio-demographic characteristics, life behaviors, and health status. Socio-demographic was measured by sex, age, education, marital status, and economic status (household income per capita, Quartile 1 was the poorest and Quartile 4 was the richest). Life behaviors included smoking status (current vs. never/past), and alcohol drinking status (current vs. never/past). Health status included chronic conditions, and functional disability. Functional disability was measured by activity of daily living (ADL), including bathing, dressing, using the toilet, continence, transferring and eating.

### Analytical strategy

**Fig. 1:**
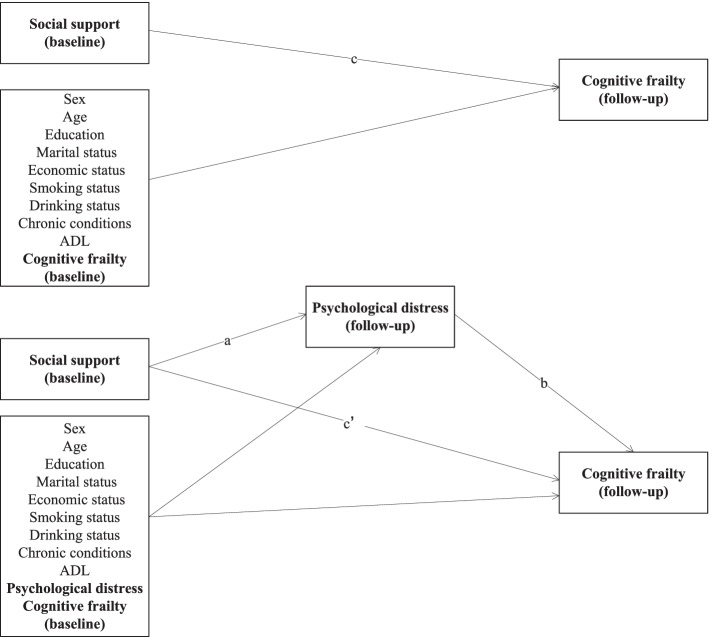
Hypothesized mediation models. Path a, the coefficient from social support to psychological distress at the follow up; path b, the coefficient from psychological distress at the follow up to subsequent cognitive frailty; path c, the coefficient from social support to subsequent cognitive frailty without psychological distress; path c’, the coefficient from social support to subsequent cognitive frailty with psychological distress

We used Chi-square tests for dichotomous variables and student t-tests for continuous variables to compare social support, psychological distress, and individual characteristics between participants with and without a cognitive frailty status at baseline survey. Path analyses with logistic function were performed to examine the direct effects of social support (predictors) on subsequent cognitive frailty (outcome) at 1-year follow-up and the mediating role of psychological distress (mediator) in this link. Both the outcome variable and mediator were measured at the 1-year follow up, and the focal parameters were labeled in Fig. 1. Specifically, we were interested in: (1) the path coefficient from social support to psychological distress at the follow-up (coefficient a), (2) the path coefficient from psychological distress at the follow-up to subsequent cognitive frailty (coefficient b), and (3) the path coefficient from social support to subsequent cognitive frailty with mediator (coefficient c′) and without mediator (coefficient c). We conducted both unadjusted and adjusted mediation models. The mediation effect was quantified as a*b [32, 33]. We also used a bootstrapping strategy resampled 5000 times to estimate the bias-corrected and accelerated 95% confidence intervals to test the indirect effects. Path analyses were performed using *Mplus* 8.3 with robust maximum likelihood estimation method, and all other analyses were performed using *Stata* 14.2.

## Results

Table 1 presents the characteristics of the respondents according to cognitive frailty at baseline. Of the 2785 respondents, the average age was 69 years and most of the older adults were female (63.55%), illiterate (41.80%), and married (75.04%). At baseline, 6.71% of participants had cognitive frailty. At 1-year follow-up, the rates of cognitive frailty were 7.47%. Participants with cognitive frailty scored significantly higher on K10 (22.41 ± 9.19 vs. 16.22 ± 7.12, t = − 11.23, *P* < 0.001) and lower on social support (39.07 ± 7.07 vs. 43.39 ± 6.11, t = 9.23, *P* < 0.001) than those participants without cognitive frailty at baseline, respectively.

**Table 1:** Sample characteristics by cognitive frailty at baseline (%)

Variables	Non-cognitive frailty (*n* = 2598)	Cognitive frailty (*n* = 187)	Total	χ^2^/*t*	*P*-value
Sex				12.15	< 0.001
Male	37.3	24.6	36.45		
Female	62.7	75.4	63.55		
Age, mean (SD)	68.97 (6.02)	72.30 (7.16)	69.19 (6.16)	−7.21	< 0.001
Educational attainment				39.97	< 0.001
Illiteracy	40.34	62.03	41.8		
Primary school	39.15	31.55	38.64		
Junior school or above	20.52	6.42	19.57		
Marital status				28.17	< 0.001
Divorced/widowed	23.79	41.18	24.96		
Married	76.21	58.82	75.04		
Economic status				17.72	< 0.001
Q1	24.13	34.76	24.85		
Q2	24.06	26.74	24.24		
Q3	25.98	24.06	25.85		
Q4	25.83	14.44	25.06		
Smoking status				0.60	0.44
Never/Past	78.91	81.28	79.07		
Current	21.09	18.72	20.93		
Alcohol drinking status				10.56	0.001
Never/Past	76.91	87.17	77.59		
Current	23.09	12.83	22.41		
Chronic conditions				7.90	0.017
No Chronic Condition	27.71	18.72	27.11		
One Chronic Condition	36.76	38.5	36.88		
Multimorbidity	35.53	42.78	36.01		
ADL disability, mean (SD)	0.30 (1.18)	1.32 (2.20)	0.37 (1.30)	−10.58	< 0.001
K10, mean (SD)	16.22 (7.12)	22.41 (9.19)	16.63 (7.44)	−11.23	< 0.001
SSRS, mean (SD)	43.39 (6.11)	39.07 (7.07)	43.10 (6.27)	9.23	< 0.001

*ADL* Activity of Daily Living Scale, *K10* Kessler Psychological Distress Scale, *SSRS* Social Support Rating Scale

Table 2 presents the unstandardized path coefficients (a, b, c’, and c) of subsequent cognitive frailty in relation to social support mediated by psychological distress. The path coefficients related to binary outcomes were presented in log-odds unit. In the unadjusted models, social support was negatively associated with psychological distress (a path in the unadjusted model: β = − 0.176, 95% CI = − 0.225 to − 0.136, *P* < 0.001) and was negatively associated with cognitive frailty (c path in the unadjusted model: β = − 0.080, 95% CI = − 0.100 to − 0.061, *P* < 0.001). The effect of social support on cognitive frailty was attenuated after adjusting for psychological distress (c’ path in the unadjusted model: β = − 0.067, 95% CI = − 0.088 to − 0.047, *P* < 0.001). Univariate analyses showed that the log-odd of cognitive frailty was significantly higher in older people who were having lower social support score. Both a*b and c’ were negative and significant when predicting from social support. A partial mediation relationship was supported for cognitive frailty, with a ratio of a*b/(a*b + c’) was 18.29%.

**Table 2:** Associations of social support and psychological distress with subsequent cognitive frailty

	Unadjusted model			Adjusted model		
Paths	Path coefficients	95% CI	$\frac{a\ast b}{a\ast b+c^{\prime }}$	Path coefficients	95% CI	$\frac{a\ast b}{a\ast b+c^{\prime }}$
SS → PD (a)	−0.176^**^	−0.225 to − 0.136		− 0.098^**^	−0.137 to − 0.066	
PD → CF (b)	0.084^**^	0.069 to 0.099		0.088^**^	0.065 to 0.107	
SS → CF (c’)	−0.067^**^	−0.088 to − 0.047		−0.028^*^	− 0.053 to − 0.007	
SS → CF (c)	− 0.080^**^	−0.100 to − 0.061		−0.040^**^	− 0.064 to − 0.016	
SS → PD → CF (a*b)	− 0.015^**^	−0.020 to − 0.010	18.29%	−0.009^**^	− 0.013 to − 0.005	24.32%

*SS* social support, *PD* psychological distress, CF cognitive frailty. Unadjusted model, predicting cognitive frailty from social support with psychological distress Adjusted model, unadjusted model + covariates in Table 1 + cognitive frailty at baseline. Path c’ controlled for the psychological distress, and path c did not control for the psychological distress

^*^p < 0.05; ^**^p < 0.01

After controlling for sex, age, education, marital status, economic status, smoking status, alcohol drinking status, chronic conditions, ADL, and cognitive frailty at baseline, we found that social support was negatively associated with cognitive frailty (path c in the adjusted model: β = − 0.040, 95% CI = − 0.064 to − 0.016, *P* < 0.001). When further adjusting for baseline psychological distress, social support was also negatively associated with psychological distress (path a in the adjusted model: β = − 0.098, 95% CI = − 0.137 to − 0.066, *P* < 0.001), and the direct effect of social support on cognitive frailty (coefficient c’) was reduced (c’ path in the adjusted model: β = − 0.028, 95% CI = − 0.053 to − 0.007, *P* < 0.001), which suggested social support played protective roles in psychological health and cognitive frailty. All path coefficients including a, b, c’ and c were statistically significant when predicting subsequent cognitive frailty from social support (the adjusted models in Table 2), suggesting that the protective effect of social support on cognitive decline may be mediated by psychological pathway. Specifically, the magnitude of direct effects of low social support on cognitive frailty changed from c’ = − 0.067 to − 0.028. The magnitude of mediation effects from social support to cognitive frailty via psychological distress changed from a*b = − 0.015 to − 0.009, and the ratio of a*b/(a*b + c’) was 24.32%. These results suggested that psychological distress partially mediated the relationship between social support and subsequent cognitive frailty, which was further confirmed by Bootstrapping tests. The results from Bootstrapping test showed that the 95% confidence interval of the indirect effect and direct effect did not span zero, indicating both the indirect effect and direct effect were statistically significant.

## Discussion

To our knowledge, this is the first study to prospectively examine the association of social support with subsequent cognitive frailty in a sample of rural Chinese older adults, as well as the mediating role of psychological distress in this process. There are two key findings. First, social support at baseline was significantly associated with decreased risk of subsequent psychological distress and cognitive frailty over 1-year follow-up. Second, the association of social support with cognitive frailty was partially mediated by psychological distress.

In this study, we also provided evidence about the prevalence of the cognitive frailty in rural China. We found the prevalence of cognitive frailty among rural Chinese older adults was approximately 7%, which was in accordance with previous studies using a similar definition in measurements (i.e. physical frailty was defined by phenotype criteria and cognitive impairment was defined by the MMSE) [30, 34]. However, one study using the data from the China Comprehensive Geriatric Assessment Study reported 2.3% of the prevalence of cognitive frailty, which is lower than our current study [35]. A possible explanation is weakness, as one of the important items in the measurement of frailty criteria, is not included in that study, which may underestimate the prevalence. Liu et al. [36] found that the prevalence of cognitive frailty is 13.3%, which is higher than the current study. One possible explanation is that Liu et al. included an older age group (≥ 65; mean age: 73) than our study. Previous studies and our research all suggest that cognitive frailty in Chinese older adults is not uncommon.

Social support is believed to be an important determinant of healthy ageing. A considerable body of epidemiological research has documented the health benefits from social support, such as lower mortality risks, better psychological and physical health outcomes [37–39]. The association between social support and cognitive frailty has been shown by a handful of cross-sectional studies [12, 13]. For example, in a study of 815 older adults aged 60 years and above in Malaysia, Malek et al. found that social support is significantly associated with decreased risk for cognitive frailty (β = − 0.021, *P* < 0.001) [12]. To our knowledge, there are no prospective studies that have specifically examined the association between social support and subsequent cognitive frailty among older people. In the current study, we found the rates of subsequent cognitive frailty over 1-year follow-up increased with lower social support (path c and c’). Social support has been considered as a trait factor that has positive consequences for health and well-being by providing people with access to various tangible, health-enhancing resources, including, but not limited to, esteem, control, and connection [40]. These psychological resources have been found to be particularly beneficial in helping people to cope with a range of challenges, such as depression and anxiety, which in turn can affect cognitive frailty. Our finding highlights again the importance of assessing and intervening in social support for older people because it is a modifiable predictor for cognitive frailty.

Our mediation analysis showed that the association between social support and cognitive frailty was mediated by psychological distress, which could explain the longitudinal association between social support and cognitive frailty in part. Specifically, older people with higher levels of social support were associated with less psychological distress (path a), which, in turn, lead to attenuated odds of cognitive frailty (path b). This suggests that people with lower social support have more likelihoods of cognitive frailty in part because they have worse psychological health. There is substantial evidence that those with higher social support have better psychological health than those with less social support [14, 41–44]. For example, Bai et al. reported social support does not promote the physical health of the Chinese elderly in rural areas, but it has a significant positive impact on their mental health [41]. Higher levels of social support mean meaningful interpersonal relations, Yang et al. found meaningful interpersonal relations may directly reduce psychological stress levels and in turn provide positive psychological implications such as enhancement of endocrine and immune functioning [45]. However, few studies have focused on the relationships between psychological factors and cognitive frailty. Only in a recent cross-sectional survey, the authors found mood disorder symptoms are strongly associated with cognitive frailty among community-dwelling people aged 60 years and over [6]. This is the first study to report psychological distress as a mediator in relationship between social support and subsequent cognitive frailty among Chinese older people. Our findings underpin the conceptual model of social relationships proposed by Berkman [46]. The model hypothesizes that health is impacted by social relationships through a series of causal processes that begin at the macro-social level (upstream factors) to micro-psychobiological processes (downstream factors). In the social network framework, psychological factors such as self-efficacy, self-esteem, depression, psychological distress, and sense of well-being represent some of the “downstream” pathways linking social relationships to health. Our study provides the evidence that social support as one of the “downstream factors” of social relationship can affect older adults’ cognitive frailty by psychological pathway (i.e., psychological distress). Psychological distress may serve as a negative form of the robust positive effects of social support on cognitive frailty in this population. Also, this finding suggests the importance of screening for psychological distress and providing strategies to mitigate the effects of poor mental health in later life. Further neurobiological and behavioral research is needed to better understand the underlying mechanisms between social support, psychological distress, and cognitive frailty among older people.

The current study has several strengths. The first strength is the longitudinal design that allows us to look at the association between social support and cognitive frailty over time. The second strength is that this study was the first to test the mediating role of psychological distress in the relation between social support and cognitive frailty among older people. The third strength is that multiple potential confounders such as socio-demographic, health behavioral (smoking and alcohol drinking), health (chronic conditions and ADL disability), and prior cognitive frailty status were controlled when examining the link between social support and cognitive frailty. Despite the strengths, our study is limited in several ways. First, the relatively small number of observations who developed cognitive frailty at the one year of follow up may cause small-sample bias. Second, the key variables (such as psychological distress) in this study, were based on self-reported data, which might lead to recall bias. Third, only one mediation variable was used in this study, and more potential paths need to be explored in the future. Finally, the current work was conducted only in rural areas, thus the results obtained from this study may be limited in urban settings, and future research should include urban areas for comparison.

## Conclusions and implications

Our study provides evidence that low social support is associated with increased rates of subsequent cognitive frailty among older people over 1-year follow-up. Furthermore, the effect of social support on cognitive frailty is partially mediated through psychological distress. These findings may have several important clinical and public health policy implications. First, the findings underscore the importance of screening older people at risk of cognitive frailty by assessing psychological distress. Second, the findings underline the need for clinicians to be alert to older people who report psychological distress, and it is important to help these older people getting appropriate treatment for their psychological distress. Third, social support is also important for older people to prevent cognitive frailty because it is a modifiable predictor, and improving the levels of social support is also a main way to effectively reduce psychological pressure for rural older people, such as organizing group activities and providing rural older service center.

## Data Availability

The datasets used in the current study are not publicly available due to them containing information that could compromise research participant privacy but are available from the corresponding author on reasonable request.

## Abbreviations

I.A.N.A: International Academy on Nutrition and Aging
I.A.G.G: International Association of Gerontology and Geriatrics
SREHC: Shandong Rural Elderly Health Cohort
MMSE: Mini Mental Status Examination
SSRS: Social Support Rating Scale
K10: Kessler Psychological Distress Scale
ADL: Activity of Daily Living
